# Investigating parental factors that lead to adolescent Internet Gaming Addiction (IGA)

**DOI:** 10.1371/journal.pone.0322117

**Published:** 2025-04-29

**Authors:** Huazhen Li, Kangzhou Peng, Yi Wu, Linna Wang, Zhanni Luo

**Affiliations:** Chongqing Normal University, **Chongqing**, China; Group for Technical Assistance / Asian College for Advance Studies, Purbanchal University, NEPAL

## Abstract

Internet gaming addiction (IGA) has become a common phenomenon that affects adolescents, due to its possible negative effects on physical and mental health issues. However, very few studies have particularly examined the relationship between adolescent game addiction and parental influences. In this study, we address some undesirable parental behaviors and aim to explore whether they influence adolescents’ internet gaming behaviors. A total of 315 adolescents who have exposed to Internet games participated in this study. We examined the relationship between four parental factors and the development process examined by the structural equation modeling (SEM) techniques: adolescent Internet gaming addiction (IGA), parental interpersonal conflict (PIC), parental loneliness (PL), parental phubbing (PP), and parental rejection (PR). We proposed nine hypotheses, five of which were supported by the data. The results suggested that parental loneliness leads to parental phubbing and rejection behaviors, as well as enhancing Internet gaming addiction among adolescents. Additionally, parental interpersonal conflict can cause parental loneliness. However, the study found that parental loneliness, parental rejection, and parental interpersonal conflict do not statistically significant impact on adolescents’ internet gaming behaviors.

## 1. Introduction

### 1.1. Definition and background

In the last decades, Internet gaming addiction (IGA) among adolescents has received much attention due to its detrimental impacts, which has become a serious public health concern [1]. Due to the quarantine and social isolation issues caused by COVID-19 pandemic, adolescents have increasingly turned to Internet-enabled devices for entertainment and education [2,3]. Spending more time at home has significantly increased their exposure to computers, which may have further heightened their interest in Internet gaming. This habit, which has persisted even after the pandemic restrictions have been lifted, has led to a more pronounced issue of gaming addiction among adolescents [4]. This alarming trend has drawn attention from public health officials, educators, and policymakers in various countries around the globe.

Internet gaming addiction can negatively influence many aspects of life, including personal well-being, academic performance, social interactions, and family relationships [5]. Individuals may experience heightened levels of anxiety, depression, and stress due to prolonged periods of gameplay, which often replaces meaningful social interactions and real-life activities. The isolation can result in feelings of loneliness and emotional instability [6]. Moreover, the nature of gaming can result in a range of physical health issues [7], including poor posture, repetitive strain injuries, and a significant reduction in physical fitness [5].

### 1.2. Gaps, significance, and research focuses

Scholars have explored the causes of Internet gaming addiction among adolescents, trying to understand the underlying factors that contribute to this growing issue. Research has identified a combination of game-related and individual factors that can drive adolescents toward gaming addiction. Game-related factors include game elements and mechanisms, such as points, badges, boss fights, and competition, which inherently engage players and encourage continuous use of video games [8–10]. On the individual side, several personal and psychological factors contribute to adolescent Internet gaming disorder (IGD), including low self-esteem, anxiety, and depression. Adolescents may turn to Internet games for escapism, finding temporary relief and a sense of achievement in the virtual world they struggle to attain in real life [11].

Scholars have proposed various strategies to address adolescent Internet gaming addiction, such as cognitive-behavioral therapy (CBT) [11] and external control therapy [5]. Among these, numerous scholars advocate for the involvement of family members in interventions, highlighting the significant role of parent-related factors in the development of adolescents’ problematic behaviors [12]. This perspective suggests that the key to solving the problem lies with those who caused it [13]. In alignment with this view, our study aims to investigate the parental factors influencing adolescent Internet gaming addiction. Through a comprehensive review of the literature, we identified four key factors: parental interpersonal conflict (PIC), parental loneliness (PL), parental phubbing (PP), and parental rejection (PR). We subsequently employed structural equation modeling (SEM) techniques to analyze the impact of these factors on adolescent IGA.

## 2. Theoretical framework and hypotheses

### 2.1. Theoretical framework

It is essential to consider various elements of parental behavior to understand the parental influence on adolescent Internet gaming addiction. Isikoglu, Erol [14] suggest parental behavior can directly impact the parent-child relationship, including parental loneliness, lack of parental emotional support [15], parental interpersonal relationship and parenting style [16], and parental knowledge of gaming and their modeling behavior [17].

Parental loneliness and parental rejection can affect the emotional atmosphere at home. If a parent feels lonely or has strained relationships, they may become emotionally unavailable or less involved, resulting in emotional neglect [18]. Additionally, poor interpersonal relationships and time spent in social activities like phubbing can cause parents to be distracted, reducing the attention they give to their children. Therefore, we categorize “parental influence” into two parts: emotional neglect and family distraction.

Parental emotional neglect has been studied previously, which may be caused by factors such as parental loneliness [19] and parental rejection [20]. A parent who often feels isolated and has few social interactions may withdraw emotionally from their child, and consistently criticize or dismiss the child’s interests.

Another major factor influencing young teenagers’ addiction to Internet gaming is family distraction. This issue is more likely to occur in families with high levels of parental interpersonal conflict [21] and parental phubbing behavior [22]. In families where parents are going through a contentious divorce, bring their work home, and spend evenings on their laptops or phones, neglecting to engage in family activities or conversations can lead to the adolescent feeling ignored and turning to Internet gaming for attention and interaction.

Hence, drawing upon prior literature, we identified four key factors that could impact Internet gaming addiction (IGA) among adolescents: parental interpersonal conflict (PIC), parental loneliness (PL), parental phubbing (PP), and parental rejection (PR).

### 2.2. Research hypotheses

#### 2.2.1. Parental rejection (PR).

Parental rejection is defined as an obstruction to adolescents receiving the fundamental care and attention they require from their parents, which typically involves a lack of emotional warmth, support or love from parents [23].

Early studies identify parental rejection as comprising behavioral and emotional rejection [15]. Behavioral rejection includes hostile and aggressive actions, such as hitting, biting, scratching, pinching, shoving, cursing, sarcasm, belittling, and making thoughtless, unkind, or cruel remarks. Emotional rejection involves being unaffectionate, indifferent, and neglectful, characterized by physical and psychological unavailability and a lack of attention to adolescents’ needs [24].

There exists a correlation between parental rejection and adolescent Internet gaming addiction (IGA). Evidence strongly implies that parental rejection leads to negative emotions and deficits in emotional regulation [25], increasing the likelihood of adopting negative skills. Previous studies [26] also demonstrate that parental rejection is a risk factor for adolescent’s IGA. Therefore, we raised the first hypothesis (H1).

H1: Parental Rejection (PR) positively predicts Adolescent Internet Gaming Addiction (IGA).

Parental rejection is also linked to various psychological and behavioral aspects of parenting. Rohner and Pettengill [15] suggest that parental rejection is associated globally with numerous expressive sociocultural factors, including the artistic traditions of particular societies and the artistic preferences of individuals. Additionally, interpersonal conflict, loneliness, and phubbing are three significant factors that contribute to parental rejection [27–29].

#### 2.2.2. Parental interpersonal conflict (PIC).

Parental Interpersonal conflict refer to verbal or physical conflicts created by disagreements between parents or other causes [30]. There are different facets of interpersonal conflict among parents, such as frequency of conflict and hostile conflict [31].

According to Cummings and Davies [32], constant conflict between parents creates a tense and negative emotional climate within the household, affecting overall family cohesion and harmony. Moreover, when children exposed to high levels of parental interpersonal conflict often experience decreased emotional security [27], leading to increased anxiety, depression, and behavioral problems. They may feel trapped in between, intensifying these negative emotions.

Previous studies, such as those by Simons, Robertson [33], have demonstrated a link between parental interpersonal conflict and parental rejection. Conflict between parents can influence their parenting practices, often leading to behaviors that adolescents perceive as rejecting. Based on this, we proposed Hypothesis 2.

H2: Parental Interpersonal Conflict (PIC) positively predicts Parental Rejection (PR).

Meanwhile, adolescents are particularly vulnerable to parental interpersonal conflict [34]. Perceived frequent and intense parental interpersonal conflict signals unstable parental relationships, which may threaten the sense of security and cause emotional distress for the youth. Adolescents frequently seek refuge in the Internet as an escape from emotional distress, which can potentially lead to the development of Internet addiction. Therefore, we proposed Hypothesis 3.

H3: Parental Interpersonal Conflict (PIC) positively predicts Adolescent Internet Gaming Addiction (IGA).

Parental interpersonal conflict can also lead to parental loneliness, as Koçak and Mouratidis [34] demonstrated in their study. Conflicts between parents can create emotional distance and reduce marital satisfaction, resulting in feelings of isolation. This emotional strain often affects both partners, diminishing their emotional availability and support for each other, thereby increasing feelings of loneliness. Hence, we proposed Hypothesis 4.

H4: Parental Interpersonal Conflict (PIC) positively predicts Parental Loneliness (PL).

#### 2.2.3. Parental loneliness (PL).

Parental loneliness refers to the subjective experience of feeling isolated, disconnected, or unsupported in the role of parenting despite being physically surrounded by family members [35, 36]. It is the emotional state in which parents feel a lack of companionship, social support, and meaningful connections.

According to Augustijn [37], parental loneliness contributes to psychological maladjustment, which can manifest as increased rejection sensitivity. This heightened sensitivity may cause parents to misinterpret or react negatively to adolescents’ behaviors, leading to more rejecting behaviors. For instance, parents who feel lonely and unsupported might become overly critical or emotionally distant, increasing the likelihood of their children feeling rejected. Consequently, we proposed the following hypothesis:

H5: Parental Loneliness (PL) positively predicts Parental Rejection (PR).

Parental loneliness may also have adverse effects on adolescents’ mental health, as lonely individuals may feel stressed by social interactions due to their lack of proficiency in handling such matters, thereby preventing adolescents from engaging in social interactions. Lonely parents, due to their difficulty in managing social relationships, may find it hard to assist adolescents in developing intimate interpersonal relationships with their peers or even with their own parents [26,38]. Adolescents with inadequate interactions with their parents attempt to meet their social needs by playing games, which promotes excessive and unhealthy Internet game use or Internet use [39]. Based on this literature, we proposed the sixth hypothesis:

H6: Parental Loneliness (PL) positively predicts Adolescent Internet Gaming Addiction (IGA).

#### 2.2.4. Parental phubbing (PP).

Parental phubbing is the act of parents frequently diverting their attention to mobile devices during family time [40], which can negatively impact the parent-child relationship and adolescents’ emotional well-being. Previous research has shown that parental phubbing is influenced by several factors, with parental loneliness being particularly significant.

Research [41] shows a significant relationship between boredom proneness and parental phubbing. Boredom proneness leads to higher levels of phubbing behavior, where parents distractedly use their smartphones during interactions with their children. This relationship is mediated by loneliness and the fear of missing out [41]. Studies indicate that boredom increases loneliness, elevating phubbing tendencies, as parents seek engagement and stimulation through their phones instead of interacting with their children. Accordingly, we establish hypothesis H7:

H7: Parental Loneliness (PL) positively predicts Parental Phubbing (PP).

Apparently, parental phubbing leads to parents spending excessive time on electronic devices, which reduces the conversations and engagement with their children. Empirical studies have shown that parental phubbing is positively associated with feelings of depression and rejection [42], indirectly contributing to parental rejection and adolescent Internet gaming addiction [43, 44]. Additionally, an increase in the time spent on technology or media leads to a reduction in the time spent on parent-child communications [23]. As suggested by Wolfers, Kitzmann [45], parental phubbing exacerbates adolescent mobile phone addiction through decreased parent-child attachment.

H8: Parental Phubbing (PP) positively predicts Parental Rejection (PR).
H9: Parental Phubbing (PP) positively predicts Adolescent Internet Gaming Addiction (IGA).

**Fig 1:**
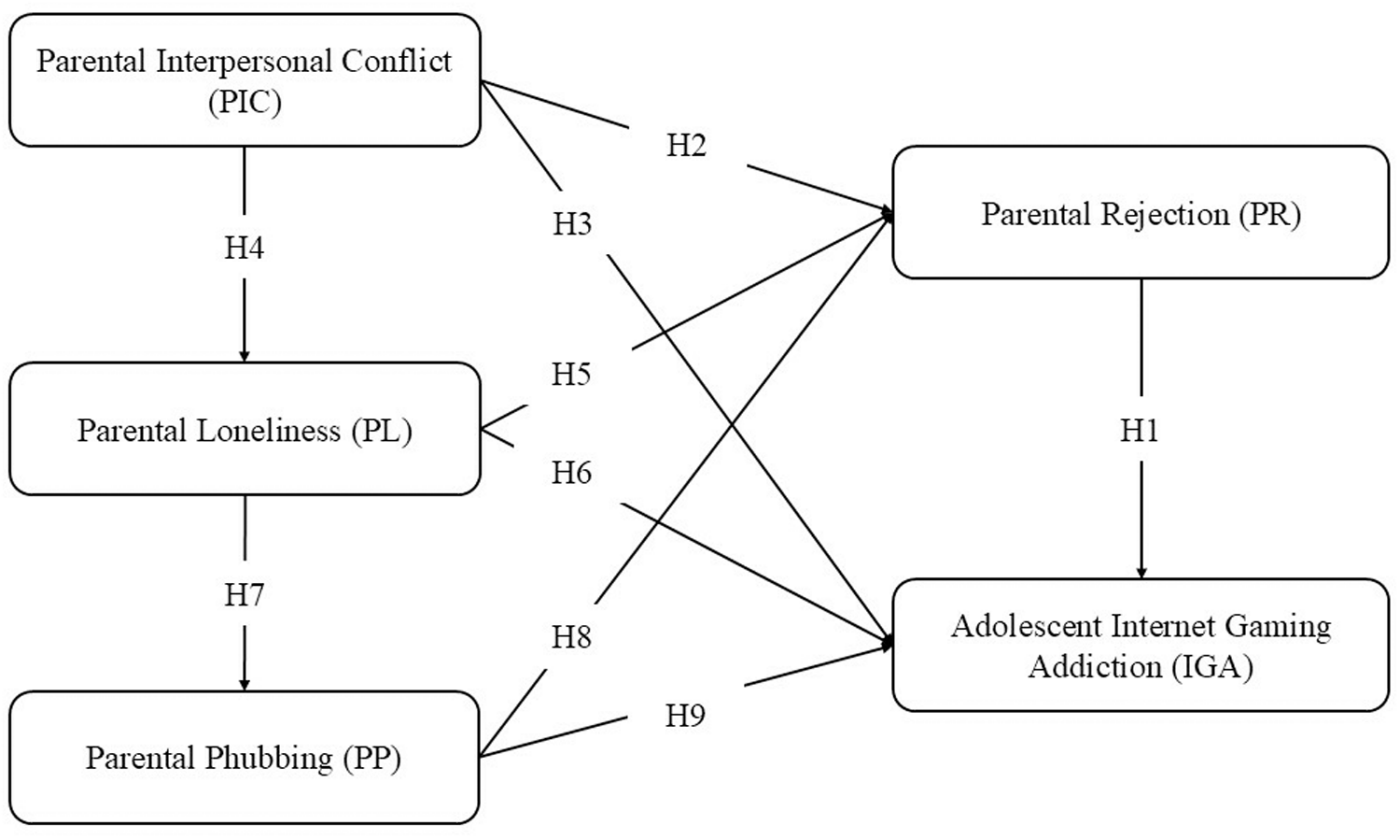
Theoretical framework and hypotheses.

The theoretical framework and hypotheses are depicted in Fig 1.

## 3. Methodology

In this research, a quantitative approach coupled with convenience sampling was employed. A range of analytical methods were utilized, including exploratory factor analysis, descriptive statistics, correlation analysis, and path analysis. A comprehensive explanation of these techniques can be found in the Findings section.

### 3.1. Research instrument

The research tool in this study was divided into two main sections. The first section, titled “Demographic Information,” was dedicated to collecting basic participant details, including gender and academic grade. The second section, named the “Parental Behaviors and Adolescents Internet Gaming Scale”, consisted of five subscales that measure five constructs: parental interpersonal conflict (PIC), parental loneliness (PL), parental phubbing (PP), parental rejection (PR), and Internet gaming addiction (IGA).

The subscale measuring parental interpersonal conflict was adapted from the Frequency, Intensity, and Perceived Threat subscales of the Family Disagreements Scale [46]. The reliability and validity of the FDS have been demonstrated in studies on parental interpersonal conflict [47,48], showing good psychometric properties.

The subscales measuring parental loneliness were adapted from the scale developed by Russell [49]. This scale includes six items, for example, “I think my parents lack companionship”. Previous studies conducted in various countries [19,50] have successfully utilized Russell’s scale, demonstrating good psychometric properties.

The subscales measuring the parental phubbing Scale were designed based on the Partner Phubbing Scale designed based on the Partner Phubbing Scale [51]. This study utilized six of the original four items (e.g., “During leisure time that my parent and I can spend together, my parent uses his/her cell phone.”).

The subscales measuring the parental rejection Scale were adapted from the Rejection subscale of the Parents as a Social Context Questionnaire [52], demonstrating good psychometric properties in several studies [23]. This scale includes 4 items designed to assess perceived parental rejection. For example, one item states: “Nothing I do is good enough for my parents.” This item reflects the perceived inadequacy felt by the respondent in their parents’ eyes, which can significantly impact their emotional well-being.

The Internet gaming addiction Scale, designed based on the Internet Addiction Scale [53]), comprises four items. This questionnaire assesses the propensity for Internet gaming addiction. For instance, one item reads: “I stay online longer than originally intended.” This statement highlights the user’s difficulty in regulating their Internet gaming time, which can greatly affect their daily life and overall well-being.

All items, except for the two demographic questions, were measured using a 5-point Likert scale, ranging from 1 (strongly disagree) to 5 (strongly agree).

### 3.2. Reliability and validity

To ensure the data is reliable, one polygraph question and four reverse-scored questions were included to identify and remove untrustworthy responses. This helps improve the quality of the thesis data. Table 1 provides details of these items.

**Table 1:** Survey items for better reliability and validity.

#	Item	Question Type
PIC5	“My parents hardly ever yell when they have a disagreement.”	Polygraph question
PIC1	“I never see my parents arguing or disagreeing.”	Reversed question
PL2	“I think my parents have a lot in common with the people around them.”	Reversed question
PL4	“I think my parents can perfectly integrated into a group.”	Reversed question
PP5	“My parent does not use his/her phone when we are talking.”	Reversed question

Participants were instructed to disagree with the polygraph question (“My parents hardly ever yell when they have a disagreement.”), and provide opposite answers to the four items (“I never see my parents arguing or disagreeing.”, “I think my parents have a lot in common with the people around them.”, “I think my parents can perfectly be integrated into a group.” and “My parent does not use his/her phone when we are talking.”).

Out of the 380 responses, 65 were removed based on these three items, resulting in 315 valid surveys. The valid response rate was 83%.

### 3.3. Participants and ethics considerations

We distributed the questionnaire to adolescents aged 16–24. The WHO defines adolescents as those aged 10–19 [54], but there is continuing debate in academia about extending this range to 24 years old. For instance, Sawyer, Azzopardi [55] suggest using 10–24 as the age range. Thus, we adopted this broader age range for our study participants.

Our study recruited 380 adolescents from China who had previously played Internet games. After excluding 65 participants (17%) due to insufficient reliability, our final sample size was 315 participants. Of the remaining 315 participants, 180 (57%) were male and 135 (43%) were female. Most participants (n = 284, 90%) were aged between 16 and 18 (see Table 2).

**Table 2:** Demographic information of the participants.

Demographic Variable		Sample (all=315)	
Number	Percentage
Gender	Male	180	57%
	Female	135	43%
Age	16 to 18 years old	284	90%
	19 to 24 years old	31	10%

Ethical protocols were observed throughout the entire data collection phase. We sent out the survey to participants, providing detailed information about the study, emphasizing that they have the autonomy to withdraw from the study at any moment, promised that the data would be utilized solely for academic research, and asked the participants to give written consent before formally taking the survey. For participants under 18 years old, consent from a parent or legal guardian was required. The process was conducted voluntarily and ensured participant anonymity. The data collection began on 24 December 2023 and concluded on 15 February 2024.

The study received approval from the Academic Research Ethics Committee (AREC) of the School of Foreign Languages and Literatures at Chongqing Normal University, with the approval number being AREC2023SFLL122302.

### 3.4. Data collection and analysis

After finalizing the questionnaire content and selecting participants, we hired a professional translator to translate it into Chinese. To ensure accuracy, we used the back-translation method. Once the translation was confirmed, we converted the questionnaire into a digital format and distributed it to students through convenience sampling. Data collection took four months.

The data analysis was conducted using IBM SPSS Statistics 26.0. and IBM SPSS Amos 26.0. Initially, Confirmatory Factor Analysis (CFA) was conducted to assess reliability and establish validity, evaluating the subscales’ fit, stability, and effectiveness. Subsequently, descriptive analysis and correlation analysis were carried out to investigate the subjects’ fundamental characteristics. Finally, Structural Equation Modeling (SEM) techniques were utilized to explore the interrelationships among the five constructs.

## 4. Findings

### 4.1. Descriptive statistics

Table 3 reports the selected variables’ mean values, standard deviation values, skewness, and kurtosis. The data reveals that the mean scores for all variables are relatively high, ranging from 3.83 to 4.09 on a scale, indicating overall positive perceptions among the respondents. The data distribution is consistently left-skewed, with values indicating. This applies to most respondents with higher values, with fewer variables stretching out to the left.

**Table 3:** Descriptive statistics.

Construct	N	Mean	Std. Deviation	Skewness	Kurtosis
PIC	315	4.07	0.773	−1.065	0.654
PL	315	3.83	0.839	−0.799	0.334
PP	315	3.91	0.880	−1.205	1.34
PR	315	3.93	0.950	−1.497	1.865
IGA	315	4.09	0.815	−1.472	2.215

### 4.2. Exploratory factor analysis

The data analysis confirms the dataset’s appropriateness for factor analysis, as indicated by a high Kaiser-Meyer-Olkin (KMO) measure of 0.921, which is well above the acceptable threshold of 0.6. This elevated KMO value suggests that the sample size is sufficient for the analysis. Additionally, Bartlett’s Test of Sphericity yields a chi-square value of 4822.172 with a p-value significantly less than 0.001, confirming the interrelatedness of the variables and supporting the use of factor analysis for this dataset.

The factor analysis was performed using Principal Component Analysis with Promax rotation. Each survey item within the constructs shows substantial factor loadings, signifying a strong correspondence with their respective constructs. The loadings vary from 0.683 to 0.801, which underscores a solid relationship between the items and their underlying factors (refer to Table 4 for details).

**Table 4:** Survey items and the exploratory factor analysis.

Construct	Item Code	Factor/Factor Loading				
1	2	3	4	5
Parental Interpersonal Conflict (PIC)	PIC1	0.801				
	PIC2	0.768				
	PIC3	0.757				
	PIC4	0.772				
	PIC5	0.793				
	PIC6	0.784				
Parental Loneliness (PL)	PL1			0.798		
	PL2			0.775		
	PL3			0.776		
	PL4			0.746		
	PL5			0.788		
	PL6			0.755		
Parental Phubbing (PP)	PP1					0.765
	PP2					0.753
	PP3					0.797
	PP4					0.744
	PP5					0.769
	PP6					0.752
Parental Rejection (PR)	PR1		0.706			
	PR2		0.717			
	PR3		0.723			
	PR4		0.741			
	PR5		0.683			
	PR6		0.685			
	PR7		0.699			
Adolescent Internet Gaming Addiction (IGA)	IGA1				0.699	
	IGA2				0.706	
	IGA3				0.709	
	IGA4				0.713	
	IGA5				0.689	
	IGA6				0.753	
	IGA7				0.744	
Total Variance Explained: 60.680%		12.42%	12.22%	12.13%	11.98%	11.92%
Cronbach’s Alpha(α): 0.908		0.897	0.866	0.889	0.856	0.879
AVE (Average Variance Extracted):		0.607	0.501	0.597	0.513	0.583
CR (Composite Reliability):		0.879	0.895	0.890	0.883	0.847

These factors account for a total variance of 60.680%, which points to a strong model fit and the constructs’ capability to explain the data variability. The distribution of this variance among the factors, with Parental Interpersonal Conflict being the most influential, underscores the varying contributions of each construct to the overall model.

We used Cronbach’s alpha to measure internal consistency reliability, which assesses how well several items in a test or questionnaire measure the same construct or idea. A Cronbach’s alpha coefficient above 0.7 is typically considered indicative of good internal consistency reliability [56]. The findings demonstrated that all five subscales’ internal consistency coefficients were above the benchmark of 0.7, indicating that they were all satisfactory. In particular, the overall survey’s Cronbach’s alpha coefficient value was 0.908, while the values for the five subscales were 0.897 (PIC), 0.889 (PL), 0.879 (PP), 0.866 (PR), and 0.856 (IGA).

Moreover, the Average Variance Extracted (AVE) for each construct exceeds the benchmark of 0.5, and the Composite Reliability (CR) values are all above 0.8, which substantiates the reliability and the construct validity of the measurement model used in the study (see Tabel 4).

### 4.3. Discriminant validity analysis

The strong positive correlation between PIC and PL implies that these factors are interdependent. This suggests that when parents fight more, they are more likely to feel lonely. Similarly, the positive correlation between PL and PP indicates that parental loneliness may be crucial in causing parental rejection of adolescents.

The square roots of AVE (ranging from 0.708 to 0.779) exceed the Inter-construct correlations, indicating discriminant validity. Furthermore, the high correlations between the constructs, such as between PIC and IGA, suggest a strong interrelationship, reinforcing the relevance and interconnectedness of these variables in the study context (see Table 5).

**Table 5:** Correlation and the square root of AVE.

Construct	AVE	Square Root of AVE	Correlation				
IC	PL	PP	PR	IGA
PIC	0.607	0.779	1				
PL	0.597	0.773	0.246***	1			
PP	0.583	0.763	0.349***	0.287***	1		
PR	0.501	0.708	0.380***	0.361***	0.319***	1	
IGA	0.513	0.716	0.239***	0.280***	0.118*	0.328***	1

* *p*<0.05

** *p*<0.01

*** *p*<0.001

### 4.4. Model fit

A variety of model-fit indices were utilized to assess the adequacy of the proposed model, encompassing χ²/df, GFI, AGFI, RMSEA, NFI, RMR, and CFI. The criteria for a good fit were set based on the recommendations of Bentler and Bonett [57], Hu and Bentler [58], and MacCallum, Browne [59], and are detailed in Table 6.

**Table 6:** Model fit indices.

Goodness-of-fit measure	χ²/df	GFI	RMSEA	RMR	CFI	NFI	AGFI
Recommend value	Between 1 and 3	>0.9	<0.08	<0.05	>0.9	>0.9	>0.9
Result	1.658	0.926	0.046	0.030	0.980	0.952	0.900

Table 6 illustrates that the proposed model has an outstanding overall fit. Notably, the model shows a superior fit in terms of χ²/df (1.658), RMSEA (0.046), RMR (0.03), NFI (0.952), GFI (0.926), and CFI (0.980). In summary, the fit indices presented in the table indicate that the model meets the established standards, thereby justifying the continuation of path analysis and hypothesis testing among the variables.

### 4.5. R-squared value

The structural equation model analysis revealed that the R-squared value for the primary dependent variable, Parental loneliness (PL) was 0.79. This indicates that 79% of the variance in parental loneliness can be explained by the predictors included in the model (see Table 7). Similarly, the R-squared values for other dependent variables such as parental phubbing (PP), parental rejection (PR), and Internet gaming addiction (IGA) were 0.83, 0.88, and 0.81, respectively.

**Table 7:** R-squared values for dependent variables.

Dependent Variables	Result	
R-Squared Value	Interpretation
Parental loneliness (PL)	0.79	Excellent
Parental phubbing (PP)	0.83	Excellent
Parental rejection (PR)	0.88	Excellent
Adolescent Internet gaming addiction (IGA)	0.81	Excellent

According to Cohen [60], an R-squared value of 0.65 can be considered large, indicating a robust model fit, so these findings suggest a substantial explanatory power for all dependent variables. The high R-squared values align with previous research in this field, further validating our model.

### 4.6. Path analysis

Table 8 depicts the path coefficients of the proposed research model, which revealed that eight out of ten hypotheses have been confirmed.

**Table 8:** Hypotheses-testing result.

#	Hypothesis	Hypothesized Path	Estimate	*p*-value		Hypothesis Testing Result
				Value	Sig.	
1	H1	PR ◊ IGA	0.232	0.232	0.232	Unsupported
2	H2	PIC ◊ PR	−0.024	0.906	0.906	Unsupported
3	H3	PIC ◊ IGA	0.121	0.496	0.496	Unsupported
4	H4	PIC ◊ PL	1.346	0.000	***	Supported
5	H5	PL ◊ PR	0.550	0.013	*	Supported
6	H6	PL ◊ IGA	0.529	0.023	*	Supported
7	H7	PL ◊ PP	0.968	0.000	***	Supported
8	H8	PP ◊ PR	0.509	0.000	***	Supported
9	H9	PP ◊ IGA	−0.088	0.468	0.468	Unsupported

*p<0.05 **p<0.01 ***p<0.001

Specifically, the results demonstrate that parental interpersonal conflict (PIC) significantly influences parental loneliness (PL) (*β* = 1.346, *p* < 0.001). Parental loneliness (PL) significantly impacts parental rejection (PR) (*β* = 0.550, *p* = 0.013, < 0.05), Internet gaming addiction (IGA) (*β* = 0.529, *p* = 0.023, < 0.05), and parental phubbing (PP) (*β* = 0.968, *p* < 0.001). Meanwhile, the data shows that parental phubbing can significantly lead to parental rejection (*β* = 0.509, *p* < 0.001). Therefore, hypotheses H4, H5, H6, H7 and H8 are sup-ported.

We hypothesized that adolescents who perceive more parental interpersonal conflict, parental rejection, and parental phubbing behavior, adolescents are more likely to develop problematic behaviors and psychological issues, leading to increased dependence on Internet games and eventually resulting in Internet gaming addiction. However, contrary to expectations, the results showed that the PIC (*p* = 0.496), PP (*p* = 0.468), PR (*p* = 0.232), and IGA are not statistically or significantly related. As a result, hypotheses H1, H3 and H9 are not supported.

Meanwhile, H2 was not validated. The results indicated that parental interpersonal conflict does not significantly impact parental rejection (*p* = 0.906). This means that conflict between parents does not cause parental rejection behavior toward adolescents. The reasons for these unexpected results are unclear and need further discussion and validation (see Table 8).

## 5. Discussion

In this study, we have developed a theoretical framework that illuminates the factors influencing adolescents’ Internet gaming addiction, encompassing parental interpersonal conflict, parental loneliness, parental phubbing, and parental rejection. We tested nine hypotheses, with five finding support in the data, while four remained unconfirmed.

### 5.1. Analysis of five supported hypotheses

The data shows that parental loneliness is a significant factor among the factors related to adolescent’s Internet gaming addiction. Parental loneliness can lead to parental phubbing, as the H7 is statistically significance (*p* < 0.001). Parental loneliness can also lead to parental rejection (H5, *p* = 0.013), meaning that parents may tend to reject their children’s real or emotional needs. This rejection can appear because lonely parents might struggle with their own emotional well-being, making it difficult for them to respond adequately to their children’s needs [61]. Additionally, the data shows that parental loneliness leads to children’s Internet gaming addiction (H6, *p* = 0.023), which is inconsistent with the previous research findings [62,63].

It is easy to understand that parental loneliness leads to parents’ excessive use of mobile phones: Parents often experience a lack of communication and a sense of isolation in their daily lives. This sense of isolation can be attributed to various factors, such as heavy work [64], the absence of a robust social support network [65], or the challenges of balancing family responsibilities [66]. Another explanation is that parents who feel lonely often turn to the Internet for a sense of belonging, as various digital platforms facilitate social connection. The design of many mobile phone applications is designed to engage users and foster attachment through features like instant notifications and personalized content. These elements capture users’ attention and enhance their emotional investment in the virtual environment. It is worth noting that the lack of parental communication is influenced by various factors, such as parents who are busy at work or those experiencing career setbacks, which affect how much they use their mobile phones. These factors should be taken into account in future research.

Parental loneliness can lead to emotional problems for both parents and children, resulting in challenges to the parent-child relationship [67]. Because feelings of isolation can lead to decreased patience and empathy, causing parents to be less attentive and responsive to their children’s requirements [68]. Moreover, parents may develop heightened sensitivity and become more critical of their children, which creates emotional distance. Consequently, the emotional disconnect can result in a situation where children, feeling neglected, may turn to Internet games as a means of coping, thereby exacerbating the issue of gaming addiction. These reasons explain why the data shows that parental loneliness can lead to parental rejection, resulting in adolescent Internet gaming addiction (H5, H6).

### 5.2. Analysis of four unsupported hypotheses

Among the four unconfirmed hypotheses, one is H2: Parental interpersonal conflict (PIC) positively predicts parental rejection (PR). The data indicates that parental interpersonal conflict does not necessarily lead to parental rejection. Gordon, Harold [30] suggest that dynamics between spouses differ significantly from those between parents and children. Parents can argue intensely with each other while still maintaining a nurturing and supportive relationship with their children. In addition, conflict between parents can be categorized into factual conflicts and emotional conflicts [34]. Factual arguments often arise from disagreements over specific issues, while emotional arguments stem from personal frustrations. The key factor is not the conflict itself but rather the underlying relationship quality and the parents’ negative emotions. However, parental conflict does not always damage the parent-child relationship or lead to parental rejection of children.

Other unconfirmed hypotheses are H1, H3, and H8. The data indicates that parental interpersonal conflict, parental phubbing, and parental rejection do not significantly and statistically relate to adolescent Internet gaming addiction. which contradicts previous research [69, 70].

One possible explanation is that children’s self-esteem and cognitive predispositions might play a more direct role in developing Internet gaming addiction [71]. For instance, low self-esteem and certain maladaptive cognitive patterns are found to be more significant predictors of gaming addiction than specific parental behaviors like phubbing or interpersonal conflict. Additionally, the subjects of the behavioral and mental issues are the parents, whereas the subjects of gaming addiction are the adolescents. The difference in subjects means that while parents may have various problems, these do not necessarily affect the children’s behavior.

Meanwhile, the result indicates parental phubbing does not lead to adolescent Internet gaming addiction, which is inconsistent with previous research. One possible reason is that our subjects are older and spend a significant amount of time at school rather than at home, thus experiencing less parental influence [72]. Specifically, in this study, we referenced the article by Sawyer, Azzopardi [55] when selecting our subjects, resulting in a sample consisting of students aged 10–24. During this period, students spend a significant amount of time at school, which greatly reduces the influence of their parents. While this group was more susceptible to parental factors such as phubbing during childhood, digital devices were not as prevalent at that time, making parental phubbing less common. Therefore, this result does not imply that parental phubbing does not lead to adolescent Internet gaming addiction but rather reflects the characteristics of our participants and the context of this study. Future research could re-examine the impact of parental phubbing on adolescent Internet gaming addiction in a context where both adolescents and parents live in an environment rich with digital devices and Internet games.

In addition, current research indicates that parental phubbing may indirectly influence children’s Internet gaming addiction via melancholy symptoms. For example, Zhou, Li [73] discovered that parental phubbing elevated depressive symptoms, raising the chance of gaming addiction. Because depressive symptoms might lead to excessive gaming as a coping mechanism, this mediation could explain the difference between our findings and previous research. However, our study did not specifically investigate this mediating role, which could explain the inconsistency.

Overall, this study emphasizes the importance of parental mental and behavioral problems in adolescents’ Internet gaming addiction. Some parent’s mental issues have been largely neglected for a long time, yet research has found that parent loneliness can indeed predict academic performance [19]. The increasing focus on how parents influence adolescents’ Internet gaming behavior has led researchers to examine the effects of parental loneliness and parental rejection on gaming addiction among teenagers from various perspectives. Specifically, researchers have attempted to categorize the impact of parental influence on adolescents’ Internet behavior into two main concepts: the effects of parental loneliness and the effects of parental rejection on gaming addiction. This division highlights the importance of considering the parent-child relationship and social dynamics of parents in understanding adolescent behavior [74]. Furthermore, scholars have differentiated these influences into various levels, examining both direct and indirect effects [75]. Parental loneliness captures the emotional state and social isolation of parents, which can impact their parenting style and subsequently affect their children’s gaming habits. Parental rejection, on the other hand, focuses on the negative emotional responses and attitudes parents may display towards their children, which can lead to increased gaming as a coping method for adolescents. This distinction recognizes that parents’ emotional and social states play a crucial role in shaping their adolescent’s Internet-using behaviors and potential gaming addiction, ultimately affecting their overall well-being and development.

Although this study indicates that parental interpersonal conflict, parental phubbing, and parental rejection have not influenced adolescent Internet gaming addiction, it suggests that other factors may influence adolescents’ Internet gaming behavior. Scholars are encouraged to explore the determinants of emotional well-being, rather than focusing solely on the impact of parental behavior. For instance, school environment, peer interactions, and personal resilience may significantly shape adolescents’ emotional health and gaming behaviors.

## 6. Conclusion

In summary, this study examines the influence of parental psychology and behavior on adolescent Internet gaming addiction. The study involves 315 adolescents who play Internet gaming addiction, examining the relationships among factors such as parental interpersonal conflict, parental loneliness, parental phubbing and parental rejection. Four out of nine hypotheses were not confirmed, suggesting that some parental mental or behavioral issues do not have a statistically significant impact on adolescents’ Internet gaming behavior.

While this study has yielded valuable insights, it is not exempt from limitations. Firstly, the majority of the participants in our investigation are college students and are of an advanced age. The advanced age of the participants might mean they have different life experiences, priorities, and perspectives compared to younger students. Younger students are more vulnerable to parental influences and family ties, which can have a substantial impact on their gaming behaviors and risk of addiction, according to research on Internet gaming addiction conducted by Brooks, Chester [76]. Future research should include a more diverse age range to better understand the varying impacts of Internet gaming addiction across different age groups.

In addition, the very small sample size within several age categories may constrict the results’ generalizability. When participants are divided into various age groups, the statistical power of subgroup analyses may be diminished, which could impact the reliability of the findings. As a result, conclusions based on smaller subsamples should be interpreted cautiously. To improve the findings’ external validity and dependability, future research should enlist bigger and more evenly distributed sample sizes from various age groups.

Furthermore, this study mainly uses a quantitative approach, which limits the depth of interpretations for the four rejected hypotheses. Although we included participant interactions to supplement these findings, they lack the thoroughness of comprehensive data. Luo [77] suggests that future studies could use a mixed sequential approach, combining qualitative and quantitative research. This method starts with quantitative analysis to explore variable relationships, followed by a qualitative study to understand the reasons behind these relationships or findings.

Additionally, different types of games may have varying psychological effects [78]. Some games have been found to help alleviate psychological stress rather than contribute to addiction. While our study focuses on Internet gaming addiction as a whole, future research could explore how different game genres influence adolescent gaming behavior.

Future research needs to consider the influence of school and peers on adolescents’ Internet gaming behaviors [79]. Since schools can implement educational programs that address the risks of game addiction and promote healthy digital habits, on the other hand, the participants in this study were adolescents aged 10–18 years, and future research could replicate this study with younger children to explore potential differences [80]. Meanwhile, as parental loneliness is a critical aspect that directly influences adolescents’ gaming behaviors, future research should also investigate how society can ease parental isolation, engage positively with their children, set a healthier example, and establish stronger family bonds [81].

Additionally, future studies should not only focus on gaming addiction but also examine problematic gaming behavior. This change in focus allows researchers to target individuals who have not yet developed severe addiction, facilitating early intervention and preventive measures [82]. Moreover, parental behavior and adolescent Internet game addiction may be influenced by cultural factors [83]. Our study was conducted in China, so its findings may not be fully generalizable to other cultural contexts. Future research could consider cross-cultural comparisons to better understand these dynamics [84].
